# The role of orexins/hypocretins in alcohol use and abuse: an appetitive-reward relationship

**DOI:** 10.3389/fnbeh.2012.00078

**Published:** 2012-11-22

**Authors:** Andrezza K. Kim, Robyn M. Brown, Andrew J. Lawrence

**Affiliations:** ^1^Addiction Neuroscience Laboratory, Behavioural Neuroscience Division, Florey Neuroscience Institutes, University of MelbourneParkville, VIC, Australia; ^2^Departamento de Psicobiologia, Escola Paulista de Medicina, Universidade Federal de São PauloSão Paulo, Brazil; ^3^Centre for Neuroscience, University of MelbourneParkville, VIC, Australia

**Keywords:** orexin, hypocretin, alcohol, relapse, reward-seeking behavior, drug-seeking, ethanol

## Abstract

Orexins (hypocretins) are neuropeptides synthesized in neurons located in the lateral (LH), perifornical, and dorsomedial (DMH) hypothalamus. These neurons innervate many regions in the brain and modulate multiple other neurotransmitter systems. As a result of these extensive projections and interactions orexins are involved in numerous functions, such as feeding behavior, neuroendocrine regulation, the sleep-wake cycle, and reward-seeking. This review will summarize the literature to date which has evaluated a role of orexins in the behavioral effects of alcohol, with a focus on understanding the importance of this peptide and its potential as a clinical therapeutic target for alcohol use disorders.

## Overview

Orexins (orexin-A, a 33-amino acid peptide, and orexin-B, a 28-amino acid peptide), or hypocretins (hypocretin-1 and hypocretin-2) are neuropeptides derived from same precursor: pre-proorexin or pre-prohypocretin (De Lecea et al., 1998; Sakurai et al., 1998) and they are the main endogenous ligands of the orexin/hypocretin system. The name *orexin* was given to the peptide because it means appetitive in Greek (Sakurai et al., 1998) and *hypocretin* is intended to indicate a hypothalamic member of incretin family (De Lecea et al., 1998). In this review we use the term orexin to describe this system and its components.

To date, two receptors have been identified for the orexin peptides: the OX-1 receptor (OX1R), which has higher affinity orexin-A; and the OX-2 receptor (OX2R), which has equal affinity for both orexin-A and -B (Sakurai et al., 1998). The orexin receptors are members of the family of seven-transmembrane G protein-coupled cell surface receptors (Sakurai et al., 1998). OX1R reportedly couples exclusively with Gq-protein whereas OX2R has been shown to couple with Gq- and Gi/o-proteins (Zhu et al., 2003).

A number of antagonists of OX1R and OX2R have been developed by different pharmaceutical companies, initially with the goal of investigating sleep disorders. In this section we describe some examples of these compounds, our list is not intended to be exhaustive, since drug development is a dynamic and changing process. GlaxoSmithKline (GSK) developed the OX1R antagonist SB 334867, which has a ~50 fold higher affinity for OX1R than OX2R (Rodgers et al., 2001; Smart et al., 2001). The same company also developed SB 408124, SB 410220, and SB 674042 which possess varying selectivity for human OX1R over OX2R (Langmead et al., 2004; Dugovic et al., 2009). GSK has also developed a dual OX1R/OX2R antagonist, SB 649868 which is currently in phase II clinical trials for sleep disorders (Scammell and Winrow, 2011). Almorexant is another dual OX1R/OX2R antagonist, developed by Actelion (Brisbare-Roch et al., 2007; Boss et al., 2009; Malherbe et al., 2009b). Merck, in turn, has developed three dual antagonists: DORA-1, DORA-5, and [(7*R*)-4-(5-Chloro-1,3-benzoxazol-2-yl)-7-methyl-1,4-diazepan-1-yl][5-methyl-2-(2*H*-1,2,3-triazol-2-yl)phenyl]methanone (MK-4305) (Bergman et al., 2008; Cox et al., 2009; Whitman et al., 2009; Winrow et al., 2010). Finally, Johnson & Johnson have developed a selective OX2R antagonist, JNJ 10397049, as has Hoffmann-La Roche with *N*-ethyl-2-[(6-methoxy-pyridin-3-yl)-(toluene-2-sulphonyl)-amino]-*N*-pyridin-3-ylmethyl-acetamide (EMPA) (McAtee et al., 2004; Malherbe et al., 2009a).

Orexin-containing neurons are found in dorsomedial (DMH), lateral (LH) and perifornical hypothalamic areas (PFA) (Peyron et al., 1998). These neurons send their projections widely to multiple brain regions in the cortex, hypothalamus, brainstem, and spinal cord (Broberger et al., 1998; Elias et al., 1998; Peyron et al., 1998; Date et al., 1999; Nambu et al., 1999; Van Den Pol, 1999). Orexins can modulate noradrenergic (Hagan et al., 1999; Horvath et al., 1999; Bourgin et al., 2000), serotonergic (Brown et al., 2001, 2002), cholinergic (Burlet et al., 2002), and dopaminergic systems (Korotkova et al., 2003), as well as the hypothalamic-pituitary-adrenal axis (Jaszberenyi et al., 2000; Kuru et al., 2000). In accordance with an extensive projection network of orexin neurons and their established role in modulating several major neurotransmitter systems, orexins are involved in numerous physiological functions: blood pressure regulation, thermoregulation, feeding behavior, neuroendocrine regulation, the sleep-wake cycle and, more recently, drug-seeking behavior (Allen and Cechetto, 1992; Stanley et al., 1996; De Lecea et al., 1998; Peyron et al., 1998; Sakurai et al., 1998; Harris et al., 2005; Lawrence et al., 2006; Aston-Jones et al., 2009).

## Addiction

Drug addiction (including alcohol dependence) is a chronic, relapsing disorder which consists of a compulsive pattern of drug-seeking and drug-taking behavior that takes place at the expense of other activities. Many people experiment with potentially addictive drugs and a minority become addicted; hence drug use alone does not inevitably lead to drug addiction. A key question in addiction research, therefore, is how do susceptible individuals make the transition from casual to compulsive drug use? Cellular and molecular effects of repeated drug use appear to produce long-term alterations in neural functions and ultimately remodel neuronal circuits. These changes may potentially explain how the risk of relapse from a drug-free state can persist for years, and also how drug-related cues are able to control behavior. In order to elucidate the mechanism by which the transition from drug use to addiction occurs, research is directed toward identifying and characterizing brain systems that mediate the rewarding effects of addictive drugs and if/how these brain systems are changed by drug use.

Harmful alcohol consumption is responsible for 2.5 million deaths annually, causing illness and injury to many more. According to the World Health Organization (WHO) 55% of the adult population has consumed an alcoholic beverage at least one time in their life. In 2005, worldwide alcohol consumption was equal to 6.13 litres of pure alcohol consumed per person aged 15 years or older (WHO, 2011). Unlike drugs such as cocaine, alcohol has many pharmacological targets, acting on various signaling pathways, ion channels, and neurotransmitter systems. Examples of molecular targets include γ-aminobutyric acid (GABA), glycine (Mihic, 1999), glutamate (Lovinger et al., 1989), serotonin (Lovinger, 1999), and acetylcholine (Narahashi et al., 1999) signaling.

Despite their varying pharmacological profiles and properties, most drugs of abuse share the common feature of acutely enhancing neurotransmission in the mesocorticolimbic system. This interaction can be direct, as in the case of psychostimulants, or indirect, as in the case of opioids, but all ultimately result in increased levels of extracellular dopamine in the terminal fields of this pathway (Di Chiara and Imperato, 1988; Wise, 1996). Different terminal regions are thought to be involved in the different aspects of addictive behavior. The nucleus accumbens is thought to be involved in the mediation of the acute reinforcing effects of drugs (Di Chiara, 2002; Volkow et al., 2002) whereas changes in the prefrontal cortex, orbitofrontal cortex, and anterior cingulate are related to the decreases in inhibitory control and hyper-responsiveness to drug-related cues (Kalivas and Volkow, 2005; Goldstein and Volkow, 2011). The amygdaloid complex and hippocampus play an important role in conditioning and learning associations between drugs and drug-related stimuli (Fuchs et al., 2005, 2007; See, 2005; Rogers and See, 2007). More recently, attention has been given to the role of neuropeptides in modulating the mesocorticolimbic system, including the neuropeptide orexin.

The function of a particular orexin neuron appears to depend on the location of the cell body; neurons from dorsomedial hypothalamic areas and perifornical hypothalamus are reportedly associated with arousal and stress, while orexin neurons from LH are suggested to be associated with reward-seeking processes for both natural and drug rewards (Harris and Aston-Jones, 2006). Moreover, other studies with varying drug challenges also support the notion that the hypothalamic orexin neurons are functionally heterogeneous (Fadel and Deutch, 2002; McPherson et al., 2007). Orexinergic neurons send projections to the ventral tegmental area (VTA) and nucleus accumbens (Fadel and Deutch, 2002). With regards the VTA, both dopaminergic and GABAergic neurons appear to be innervated, albeit somewhat sparsely (Balcita Pedicino and Sesack, 2007). Nevertheless, evidence is accruing for direct orexinergic input onto VTA dopaminergic cells that innervate the prefrontal cortex and nucleus accumbens (Vittoz et al., 2008). While the VTA receives input from both medial and lateral orexinergic neurons (González et al., 2012), in terms of opiate dependence, orexin inputs onto caudal VTA cells are implicated (Richardson and Aston-Jones, 2012). Thus, given the innervations of mesolimbic regions by orexin and the presence of orexin receptors in these nuclei, a framework exists to suggest a potential role in mediating the rewarding effects of drugs of abuse including alcohol. The main goal of this present review is to focus on orexins and alcohol, and to provide an update on this rapidly developing field (Lawrence, 2010).

In electrophysiological studies on brain slices bath application of orexin-A potentiates NMDA currents in VTA neurons, while orexin-B potentiates both NMDA and AMPA currents (Borgland et al., 2006). When both orexin peptides are applied, the effect is greater than seen with orexin-B alone, suggesting that both peptides exert actions through distinct receptors, signal transduction pathways and/or cellular targets (Borgland et al., 2008). Orexins caused an increased in firing frequency, burst firing, or no change in firing in different groups of A10 dopamine and non-dopamine neurons in the VTA (Korotkova et al., 2003). Microdialysis studies showed increased dopamine and metabolite levels in nucleus accumbens after intra-VTA microinjection of orexin-A and orexin-B (Narita et al., 2006).

## Orexins and alcohol self-administration

There is no apparent difference in the density of expression of the mRNA encoding pre-proorexin in the hypothalamus of alcohol-preferring P rats compared to non-preferring NP rats (Lawrence et al., 2006). It has been known for some time that High saccharin intake (HiS) rats voluntarily consume more ethanol compared to Low Saccharin intake (LoS) rats, as well as showing other addiction-prone behaviors (Dess et al., 1998). More recently, it has been demonstrated that HiS rats possess a higher number of positive orexin cells in the LH and PFA than LoS rats (Holtz et al., 2012). Rats with high novelty-induced activity show both high ethanol consumption and elevated orexin mRNA expression in the perifornical hypothalamus, while expression of orexin was reduced in rats with high triglycerides despite similar ethanol intake (Barson et al., 2012). Therefore, while there is no clear link between orexin expression and alcohol preference per se, chronic ethanol consumption does alter the expression of pre-proorexin mRNA in the LH of rats (Lawrence et al., 2006; Morganstern et al., 2010).

The operant self-administration procedure is a widely accepted animal model to study drug reinforcement and drug-seeking. Evidence for a role of orexins in the reinforcing properties of alcohol was established by the observation that OX1R-selective antagonist SB 334867 attenuated operant self-administration of ethanol by Indiana-preferring (iP) rats (Lawrence et al., 2006). Subsequently, a similar effect was demonstrated in Long–Evans rats which was specific to ethanol, as sucrose self-administration was not affected (Richards et al., 2008). This contrasts with another study which showed that 30 mg/kg of SB 334867 decreased responding for both sucrose pellets and ethanol in rats (Cason et al., 2010). More recently we have shown that while 5 mg/kg of SB 334867 can attenuate self-administration of both sucrose and ethanol solutions, there is a significantly greater effect for ethanol compared to sucrose (Jupp et al., 2011a). Moreover, we showed a greater role for OX1R in the motivational properties of ethanol, as the same dose of SB 334867 attenuated responding on a progressive ratio schedule for ethanol but not sucrose (Jupp et al., 2011a). The apparently contradictory effects observed with natural reward may relate to differences in the reinforcement schedule used in each study (FR1 vs. FR3 vs. PR), and/or the doses of compounds used. Thus, attenuation of sucrose self-administration occurs at higher doses of SB 334867 (Cason et al., 2010), where selectivity between OX1R and OX2R may be reduced.

Collectively however, these data point to a role for the OX1R in the rewarding and motivational properties of ethanol. Importantly however, a recent study found no impact of OX1R antagonism on ethanol self-administration by Wistar rats with the OX1R antagonist SB-408124 (Shoblock et al., 2011). The level of ethanol self-administration by the Wistar rats in this study was relatively low compared to alcohol-preferring strains. Hence, the authors raise the possibility that activity at OX1R is only recruited during high levels of ethanol intake, or only involved during high motivation to consume ethanol. This hypothesis is supported by the observation that SB 334867 reduces alcohol consumption and preference in Sprague–Dawley rats with a high baseline preference for alcohol while having no effect on those with a low baseline preference (Moorman and Aston-Jones, 2009). In addition, OX1R activation has been linked to high effort work for extremely salient rewards (Borgland et al., 2009).

Nevertheless, a recent study has implicated the relatively less-studied OX2R in ethanol reward. The OX2R antagonist, JNJ 10397049, reduced ethanol, but not saccharin, self-administration after systemic administration (Shoblock et al., 2011). Indeed, it has been suggested that studies with relatively high doses of “selective” OX1R antagonists may in fact be difficult to interpret as solely acting through OX1R due to loss of selectivity *in vivo* (e.g., Shoblock et al., 2011; Gotter et al., 2012). In this regard however, as we canvassed in a recent study from our lab (Jupp et al., 2011a), SB 334867 can reduce alcohol self-administration in rats at doses (5 mg/kg ip) where OX1R selectivity is retained. On balance, it appears possible that both OX1 and OX2 may play a role in mediating the reinforcing properties of alcohol (and other drugs). It would be worth addressing whether both OX1R and OX2R are implicated in natural and drug rewards more fully. This is now a real possibility with commercially available antagonists for OX1R and OX2R. In this regard, two highly selective compounds namely GSK1059865 (OX1R) and JNJ1037049 (OX2R) have been assessed in conjunction with fMRI in rat brain after an acute amphetamine challenge (Gozzi et al., 2011). The findings suggested that the OX2R effect was predominantly cortical (arousal) while the OX1R effect was predominantly subcortical striatal (reward and goal-directed behaviors). Importantly, these conclusions do not preclude a potential cortical action of OX1R. Such functionally topography may relate to suggestions that orexin projections from the LH control drug-seeking behavior through activation of reward pathways and perifornical-dorsomedial areas of hypothalamus control interface arousal and the stress pathways (Harris and Aston-Jones, 2006). Future studies will undoubtedly address the issue of the precise functional interplay between medial and lateral orexinergic populations.

The above-mentioned data relate to studies using systemic administration of drugs to manipulate the orexin system. While of value, they do not provide evidence for anatomic loci of action where orexins may be acting in the brain to regulate self-administration of alcohol. Microinjections of orexin-A into the paraventricular nucleus and LH increase ethanol self-administration, but not food or water consumption in Sprague–Dawley rats (Schneider et al., 2007). In contrast, intra-nucleus accumbens microinjections of orexin-A had no effect on ethanol intake (Schneider et al., 2007). More recently, intra-LH injections of NMDA stimulate both ethanol intake and expression of orexin in rats (Chen et al., 2012). Therefore, while hypothalamic sites where orexins regulate ethanol intake have been identified, there is still a need to fully evaluate potential extra-hypothalamic sites of action.

## Orexins in alcohol relapse/reinstatement

The extinction-reinstatement paradigm is a commonly used animal model of relapse and is used to investigate the neurobiology underlying drug-seeking behavior (Stretch et al., 1971; Davis and Smith, 1976; De Wit and Stewart, 1981). In 2005 it was reported that intracerebroventricular administration of orexin-A reinstated extinguished responding on a cocaine-paired lever in rats (Boutrel et al., 2005; Wang et al., 2009). The same has also been found for nicotine-seeking behavior in mice (Plaza-Zabala et al., 2010). SB 334867 attenuates reinstatement induced by a cocaine- and heroin-related stimulus, without affecting that induced by a stimulus conditioned to a conventional reinforcer (Smith et al., 2009; Martin-Fardon et al., 2010; Smith and Aston-Jones, 2012). A similar result is found when drug-seeking is assessed after 1 day or 2 weeks of abstinence (without extinction) or following extinction of cocaine-seeking in a different context (Smith et al., 2010). Zhou et al. (2012) showed that SB 334867 regulated cue-induced reinstatement of cocaine-seeking in male, but not female, rats.

In the alcohol field, Lawrence et al. (2006) provided the first evidence linking the orexin system in cue-induced reinstatement of alcohol-seeking. Shortly afterwards these functional data were supported by demonstration that cue-induced reinstatement of alcohol-seeking was associated with activation of orexin-containing neurons (Dayas et al., 2008). Also, in the renewal paradigm of alcohol-seeking there is a significant positive correlation between the activation of orexin neurons and relapse responding on the “active” nose-poke (Hamlin et al., 2007). Subsequently this evidence has been expanded to include the observation that SB 334867 abolishes cue-induced reinstatement of alcohol-seeking both immediately after extinction as well as after an extended period of abstinence following extinction (Jupp et al., 2011b). Collectively, these data suggest that despite extinction and long-term abstinence, the orexinergic system is seemingly still involved in the integration of the salience of cues that previously signaled the availability of ethanol. The latter study included an examination of the pattern of neuronal activation following immediate vs. delayed reinstatement. Thus, the putative anatomic loci where SB-334867 may be acting (directly or indirectly) to regulate relapse-like alcohol-seeking seemingly includes prefrontal cortical structures. Intriguingly, the putative anatomic loci where SB 334867 may act (directly or indirectly) to regulate relapse-like alcohol-seeking apparently shifts from orbitofrontal/medial prefrontal cortex, accumbens core and basolateral amygdala (BLA) following immediate reinstatement to primarily a cortical locus following delayed reinstatement. These findings collectively suggest that the circuitry through which orexin impacts upon alcohol-seeking driven by exposure to cues may change over time. Further studies, including functional mapping, are required to determine the precise loci where OX1R may regulate cue-induced alcohol-seeking, including cortical sites and subcortical targets, such as the VTA which has recently been implicated in cue-driven cocaine-seeking (James et al., 2011). Reversible inactivation of LH prevented cue-induced reinstatement of beer and sucrose-seeking rats (Marchant et al., 2009). Using the retrograde neuronal tracer combined with Fos activation method, recruitment of LH-projecting neurons from nucleus accumbens shell during reinstatement was demonstrated (Marchant et al., 2009).

Dhaher et al. (2010) reported that SB 334867 prevented the increase of ethanol consumption observed on relapse following abstinence, but did not have any effect on Pavlovian spontaneous recovery of alcohol-seeking after 2 weeks abstinence. Nevertheless, the same OX1R antagonist reduces stress-induced reinstatement to alcohol or sucrose-seeking by yohimbine (Richards et al., 2008); or the cue-induced reinstatement of alcohol seeking facilitated by intra-lateral hypothalamic injection of neuropeptides S (NPS) in rats (Cannella et al., 2009). As is the case with ethanol consumption, recent studies have found that SB 334867 is more effective at reducing reinstatement of alcohol-seeking compared to that for a natural reward (Martin-Fardon and Weiss, 2012). Interestingly, inhibition of hypothalamic peptides, including orexin neurons, by projections from nucleus accumbens shell has been implicated in the extinction of alcohol-seeking behavior (Millan et al., 2010), suggesting a possible role for orexins in extinction.

## Orexins in alcohol conditioned place preference

A study utilizing the CPP paradigm provided the first evidence for a role for the orexin neurons of the LH in reward and reward-seeking. Conditioned animals which show a preference for a reward-paired chamber (with food, morphine, or cocaine) display increased Fos expression in lateral hypothalamic orexin cells (Harris et al., 2005). It was demonstrated that neurons that project from rostral lateral septum to LH are activated in proportion to cocaine-induced CPP, and the inhibition of lateral septum neurons blocked Fos expression in orexin cells in the LH (Sartor and Aston-Jones, 2012). Studies involving functional topography of orexin projections to VTA showed that this region is a significant target of orexin action (Richardson and Aston-Jones, 2012). Administration of the OX1R antagonist SB 334867 intra-VTA was found to reduce morphine CPP (Narita et al., 2006) while administration of morphine systemically or orexin-A into the VTA reinstated this CPP (Harris et al., 2005). In addition, microinjection of Y4 receptor agonist, rPP (rat pancreatic polypeptide), into the LH reinstated the extinguished morphine CPP which could be blocked by previous OX1R antagonist treatment (Harris et al., 2005). Subsequent studies have further supported a role for OX1R in opiate place preference (Sharf et al., 2010).

Alcohol CPP studies are equivocal at this stage and it appears that there may be a differential role for OX1R and OX2R in the conditioned rewarding effects of ethanol. OX1R antagonism by SB 334867 attenuated a weak CPP to ethanol in DBA mice; however, when a different protocol which resulted in a strong CPP was utilized SB 334867 had no effect (Voorhees and Cunningham, 2011). More recently, a different OX1 receptor antagonist SB-408124 was found to have no effect on the acquisition or expression of an ethanol-induced CPP in DBA mice (Shoblock et al., 2011). These authors found that the OX2R antagonist JNJ 10397049 attenuated the acquisition, expression, and reinstatement of an ethanol-induced CPP (Shoblock et al., 2011).

## Orexins and behavioral sensitization

Behavioral sensitization has been proposed to occur as a result of changes in the neural circuits that regulate the attribution of incentive salience to stimuli (Robinson and Berridge, 2008). Intracerebroventricular injections of orexin A or orexin B increase locomotor activity in mice (Narita et al., 2006). Both systemic and intra-VTA administration of SB 334867 blocks the development of behavioral sensitization to cocaine in rats (Borgland et al., 2006). Importantly, intra-VTA SB 334867 itself does not alter locomotor activity at doses that regulate cocaine-seeking (James et al., 2011). In contrast to cocaine, SB 334867 had no effect on either acute or sensitized responses to morphine (Sharf et al., 2010). OX1R antagonism decreases ethanol-induced locomotor activity in mice, but not spontaneous activity in mice (Voorhees and Cunningham, 2011) or rats (Richards et al., 2008). In addition, recent data suggest that OX2R antagonism can attenuate ethanol-induced locomotor activity in mice (Shoblock et al., 2011). As yet, a role for orexin in behavioral sensitization to ethanol has not been thoroughly examined. However, pre-proorexin knockout mice showed intact acute locomotor responses and behavioral sensitization to morphine (Sharf et al., 2010).

## Orexins and motor function

When combined with ethanol, Almorexant, a dual orexin receptor antagonist, did not interfere in rotarod performance and grip strength, showing no action in motor coordination (Steiner et al., 2011). Moreover, this study also suggested that Almorexant has no effect upon ethanol-induced sedation. In humans, there is no potentiation of alcohol impairment by Almorexant (Hoch et al., 2012). In rats, orexin-A or -B given icv reduced the hypnotic effects of ethanol (Jia et al., 2011). In a prenatal alcohol exposure procedure, it was observed that rats which received alcohol intra-gastrically twice daily on postnatal days PD 4–9, and were then treated with SB 334867 daily on PD 12–14, presented lower levels of activity upon test than rats who had received vehicle on PD 12–14 (Stettner et al., 2011). This finding implicates the orexin system in the persistent hyperactivity observed following prenatal alcohol exposure. Intriguingly, prenatal alcohol exposure (via dams gavaged from E9 to parturition with 1 or 3 g/kg of ethanol) stimulates neurogenesis, including that of orexinergic neurons, which may contribute to altered consumption patterns in exposed offspring (Chang et al., 2012).

## Orexins and withdrawal

Somatic signals of nicotine-induced withdrawal were attenuated with OX1R antagonism or in pre-proorexin knockout mice. In parallel, nicotine withdrawal-induced Fos expression in the DMH/perifornical region and the PVN was attenuated by pretreatment with SB 334867 (Plaza-Zabala et al., 2012). In terms of ethanol, data from alcohol-dependent subjects implicates orexin in affective dysregulation that accompanies withdrawal (Von Der Goltz et al., 2011). Indeed, there is a significant association between orexin A levels and severity of withdrawal scores (Bayerlein et al., 2011).

## Future directions

In this review we have summarized recent studies focusing on implications of the orexin system in alcohol use and abuse. There are still numerous questions that remain to be answered: for example, defining the anatomic loci for putative actions of OX1R versus OX2R in regulating alcohol use, and evaluating the possibility of dual OX1/OX2R antagonists as potential pharmacotherapeutics. In relation to medication development, assessing the functioning and responsivity of orexin systems in states of alcohol dependence are also warranted. Another issue is elucidating the precise role orexins play in the conditioned reinforcing effects of alcohol across different species. From a neurobiological perspective, the integration of orexin-containing neurons into the circuitries underpinning many of the behaviors discussed requires more thorough attention. Related to this point is a pressing need to delineate the interactions between orexins and other peptides, such as NPS (Cannella et al., 2009), galanin (Karatayev et al., 2010) plus undoubtedly others, and non-peptides, such as histamine (Jia et al., 2011). While much of the data to date address the issue of a direct role for orexins in alcohol use and relapse, the field should also be cognizant of the evidence of a possible role for orexins in alcohol withdrawal. As mentioned above, recent human studies suggest that the more severe alcohol withdrawal symptoms are, the greater reduction in orexin-A expression is observed, at least in lymphocytes (Bayerlein et al., 2011). This finding, including observations on orexin A promoter methylation (Bayerlein et al., 2011), suggest the potential for involvement of orexins in aspects of the withdrawal syndrome following chronic alcohol, and possibly other drug, use. Naturally, this supposition is based upon the assumption that epigenetic alterations in lymphocytes are mirrored in neurons, which remains to be clarified. Nevertheless, our laboratory has recently demonstrated that even following protracted abstinence, orexin signaling is activated upon re-presentation of cues previously paired with alcohol availability. Therefore, orexins may actually exert multiple roles in relation to alcohol use and abuse, ranging from consumption, motivational strength, withdrawal through to relapse. Given the various lines of evidence for these factors presented within this review, it is clear that substantial further research is required to confirm or refute these hypotheses.

### Conflict of interest statement

The authors declare that the research was conducted in the absence of any commercial or financial relationships that could be construed as a potential conflict of interest.
